# The mechanisms of energy balance between oxidative phosphorylation and glycolysis in human ovarian granulosa cells

**DOI:** 10.3389/fphys.2026.1715312

**Published:** 2026-02-16

**Authors:** Jing Wang, Gai-Jing Wang, Ling-Chao Wang, Lu-Lu Wang, Jin-Jin Qin, Bei Wang, Jie Cui, Hong-Li Wu, Rui Li, Wei Liu

**Affiliations:** 1 Department of Obstetrics and Gynecology, Affiliated Hospital of Hebei University, Baoding, Hebei, China; 2 Medical Company of PLA, Baoding, Hebei, China

**Keywords:** energy metabolism, glycolysis, mitochondrial function, mural granulosa cells (mGCs), oxidative stress

## Abstract

**Objective:**

This study investigated the interplay between mitochondrial oxidative phosphorylation and glycolysis in human ovarian granulosa cells by analyzing changes induced by the mitochondrial inhibitor carbonyl cyanide m-chlorophenyl hydrazone (CCCP) and the glycolysis inhibitor bromopyruvic acid (BA).

**Methods:**

Mural granulosa cells (mGCs) were isolated from tubal factor infertility patients and cultured. Cells were divided into control, CCCP (10 µM), and BA (0.5 mM) groups. Mitochondrial function was assessed via mitochondrial membrane potential (MMP), reactive oxygen species (ROS), and adenosine triphosphate (ATP) levels. Glycolysis-related gene expression (HIF-1α, GLUT1, LDHA, PFKP) was measured by qRT-PCR after CCCP treatment. Glucose consumption and cell viability (CCK-8 assay) were evaluated in control and CCCP groups at 24, 48, and 72 h.

**Results:**

Compared to controls, both CCCP and BA groups exhibited significantly decreased in MMP (*P* < 0.0001). and ATP levels (*P* ≤ 0.001), while ROS showed a non-significant increase (P > 0.05). CCCP treatment significantly upregulated mRNA expression of HIF-1α, GLUT1, LDHA, and PFKP (*P* < 0.05). Glucose consumption rate significantly increased in the CCCP group at all time points (*P* < 0.05), peaking at 72 h. Cell viability progressively improved with longer culture duration after-CCCP treatment, showing significant increases at 48 and 72 h (*P* < 0.05), reaching levels comparable to controls by 72 h (*P* > 0.05).

**Conclusion:**

Human mGCs utilize both oxidative phosphorylation and glycolysis for energy metabolism. Inhibition of one pathway triggers compensatory upregulation of the other, indicating collaborative regulation between these pathways to maintain cellular function and follicular homeostasis.

## 1 Introduction

An ovarian follicle, the fundamental unit of the ovary, is composed of an oocyte encased by multiple layers of granulosa cells. The process of follicular development and maturation involves four stages: initiation and recruitment of primordial follicles; development of preantral follicles; selection of dominant follicles; and finally, ovulation and formation of the corpus luteum (Spanel-Borowski, 2012). During these stages, under the action of gonadotropin (Gn), the diameter of the follicle increases, and granulosa cells undergo proliferation and functional differentiation.

Studies have demonstrated that the addition of human chorionic gonadotropin (hCG), pregnant mare serum gonadotropin (PMSG), follicle-stimulating hormone (FSH), estradiol (E_2_), and human menopausal gonadotropin (hMG) to the *in vitro* maturation (IVM) medium for human oocytes does not yield the anticipated effects on oocytes lacking granulosa cells. This limitation arises because the receptors for these molecules are located on granulosa cells, highlighting the necessity of granulosa cells for oocyte development. Jahromi et al. (2015), and Araújo et al. (2014) noted that granulosa cells surrounding the oocyte promote oocyte differentiation and maturation by proliferating and supplying energy. Research by Zhang et al. (2017), and Alvarez et al. (2016) showed that granulosa cell dysfunction and abnormal energy metabolism are significant factors contributing to impaired follicular development and decreased fertility in women. Therefore, the development and maturation of oocytes are directly influenced by the growth status of ovarian granulosa cells.

Mitochondrial membrane potential (MMP) is a key indicator of cell viability. The reactive oxygen species (ROS) generated during the process of oxidative stress (OS) can compromise mitochondrial function and disrupt normal cellular function through lipid peroxidation, protein oxidation, and DNA damage. Excessive oxidative stress responses in granulosa cells during the ovarian developmental cycle can lead to a decrease in MMP and even apoptosis in severe cases. The relationship between impaired mitochondrial function in ovarian granulosa cells and the energy metabolism of these cells remains inconclusive. Nevertheless, impaired mitochondrial function in ovarian granulosa cells may be a contributing factor to the decline in oocyte developmental potential (Cecchino et al., 2021). The precise causes of follicular maturation disorder and decreased oocyte developmental potential are yet to be definitively determined. We hypothesize that impaired energy metabolism in ovarian granulosa cells might be one of the mechanisms underlying the decreased developmental potential of oocytes.

## 2 Materials and methods

### 2.1 Study participants

Female patients with infertility caused solely by tubal factors and who required *in vitro* fertilization-embryo transfer (IVF-ET) were selected for this study. All the participants were married women under the age of 35 who desired to have children, met the diagnostic criteria for infertility according to the Chinese Expert Consensus on the Diagnosis and Treatment of Tubal Infertility (2018 Edition), and were scheduled to undergo assisted reproductive technology (ART) treatment. This study received the approval of the Ethics Committee of the hospital. All patients provided informed consent in writing.

The inclusion criteria were as follows: (1) patients aged <35 years; (2) patients with a body mass index (BMI) ranging between 18 and 28 kg/m^2^; (3) patients with no clinical manifestations of hyperandrogenism; (4) patients with a regular menstrual cycle (26–32 days); (5) patients whose basal endocrine levels measured on the second–third day of the menstrual cycle were within the following range: serum follicle-stimulating hormone (FSH) < 10 IU/L; estradiol (E_2_) < 50 pg/mL; testosterone (T) 0.7–3.1 nmol/L; and prolactin (PRL) 5–25 ng/mL; and (6) patients with no history of surgery on either ovary.

The exclusion criteria were as follows: (1) patients with congenital malformations of the reproductive organs (such as unicornuate uterus, bicornuate uterus, etc.); (2) patients with other concurrent chronic diseases or endocrine disorders affecting metabolism (such as thyroid dysfunction and hyperprolactinemia); (3) patients with malignant tumors of the reproductive system; (4) patients with chromosomal abnormalities in themselves or their partners; (5) patients with allergic constitutions; (6) patients with psychiatric disorders; and (7) patients who had undergone hormone or immunosuppressive agent therapy within 3 months prior to enrollment in the study.

### 2.2 Collection of mural granulosa cells

A total of 68 patients with tubal infertility were enrolled in the study. Patients who underwent IVF-ET by controlled ovarian hyperstimulation (COH). The GnRH agonist (Ferring, Germany) was applied for approximately18 days, when the downregulation standard was reached (FSH <5 mIU/mL, LH < 5 mIU/mL, E2 < 50 pg/mL, endometrial thickness <5 mm), and Gn was subcutaneously injected until the HCG day. On the day of egg retrieval, follicular fluid (FF) collected from the patients enrolled in the study, and 10 samples with significant blood contamination were discarded. The mixed follicular fluid was centrifuged at 2,000 rpm for 10 min at room temperature. The supernatant was removed by aspiration, and the cell pellet was retained. Three ml of phosphate-buffered saline (PBS) was added to the cell pellet and gently pipetted up and down to resuspend the cells. A sterile 10 mL centrifuge tube was prepared, and 3 mL of human lymphocyte separation solution was added. The granulosa cell suspension was then gently layered onto the human lymphocyte separation solution at a 1:1 volume ratio.

After centrifugation at 2,000 r/min for 20 min, the following four distinct bands were visible in the tube, from top to bottom: a supernatant layer (light straw yellow), a granulosa cell layer (white), a separation medium layer (colorless), and a red blood cell layer.

The white granulosa cell layer was slowly and gently aspirated using a Pasteur pipette, transferred to another 10 mL sterile centrifuge tube, added with 2 mL of PBS buffer, blended thoroughly, centrifuged at 2,000 r/min for 5 min, and the supernatant was removed.

The resultant solution was treated with 2 mL of 4 °C red blood cell lysis buffer, mixed gently, left to stand for 2 min, centrifuged at 2,000 r/min for 5 min, and the supernatant was discarded. The product was added to 2 mL of PBS buffer, mixed gently, centrifuged at 2,000 r/min for 5 min, and the supernatant was removed. This process was repeated two more times.

The resultant solution was added to 1 mL of trypsin digestion solution (Trypsin Solution, Solarbio) containing 0.25% trypsin and 0.02% ethylenediaminetetraacetic acid (EDTA), gently pipetted up and down, and placed at room temperature for 1 minute. Subsequently, the solution was added to Dulbecco’s Modified Eagle Medium (DMEM)/F12 cell culture medium containing 10% fetal bovine serum to terminate the digestion. Then, the solution was centrifuged at 2,000 r/min for 10 min. The supernatant was removed and rinsed twice.

Consequently, granulosa cells from 58 independent donors (N = 58) were ultimately used for all subsequent experiments.

### 2.3 Culture of mural granulosa cells

The granulosa cell pellet was resuspended in DMEM/F12 cell culture medium containing 1% penicillin/streptomycin and 10% fetal bovine serum. Cell counting was performed using trypan blue staining, and the cell density was adjusted to 2 × 10^5^/mL viable cells/mL. The cells were then seeded into 6-well plates, with each well containing 2.5 mL of the culture medium (granulosa cells appeared round and were distributed either individually or in clusters when observed under the microscope). After seeding, the cells were incubated at 37 °C in a 5% CO_2_ incubator for 24 h, after which the medium was completely replaced.

The morphology of granulosa cells was observed under an optical microscope, confirming that the cells were growing well. The cells were then digested and seeded into 96-well plates and 6-well plates at densities of 2 × 10^4^ cells/mL and 2 × 10^5^ cells/mL, respectively. After 24 h of *in vitro* culture, the cells adhered to the plates. Following appropriate drug treatment, the culture medium and granulosa cells were collected for subsequent molecular biology assays.

### 2.4 Changes in mitochondrial functions in granulosa cells after medication administration

#### 2.4.1 Detection of mitochondrial membrane potential of granulosa cells

Principle: JC-1 is an ideal fluorescent probe for detecting MMP in cells. When MMP is high, JC-1 aggregates in the mitochondrial matrix, forming polymers that emit red fluorescence with a maximum emission wavelength of 590 nm upon excitation at 488 nm. Conversely, when MMP is low, JC-1 polymers decompose into JC-1 monomers, which emit green fluorescence with a maximum emission wavelength of 527 nm upon excitation at 488 nm. Thus, changes in MMP can be determined by observing changes in fluorescence color. The degree of mitochondrial depolarization is typically measured by the relative ratio of red to green fluorescence intensity.

1. Preparation of the JC-1 staining working solution: The 200X JC-1 stock solution was diluted with ultrapure water at a ratio of 50 μL stock solution to 8 mL ultrapure water. The resultant solution was vigorously shaken to ensure that the JC-1 was completely dissolved. Subsequently, the resultant solution was added to 2 mL of 5X JC-1 staining buffer and thoroughly mixed, obtaining the JC-1 staining working solution.
2. The granulosa cell suspension was seeded into 96-well culture plates at a cell density of 2 × 10^4^ cells per well and a suspension volume of 100 μL per well. After incubating at 37 °C in a 5% CO_2_ environment for 24 h, cell adhesion to the well was confirmed, and the culture medium from the wells was discarded. The granulosa cells were divided into three groups: the control group, the CCCP group, the BA (0.5 mM) group. After the treatment of GCs with glycolysis inhibitor BA, the ATP content was signiffcantly reduced in all GCs, especially when the concentration of BA is 2.5 mM, this concentration leads to an increase in cell mortality rate, Therefore, it was not chosen (Kansaku et al., 2017).
3. A culture medium containing mitochondrial inhibitor CCCP (10 μM) and glycolysis inhibitor BA (0.5 mM) was added to each well at a volume of 100 μL. The cells were exposed to the drugs for 20 min, and the culture medium in each well was discarded after the drug reactions. The cells were rinsed twice using PBS buffer to eliminate the effect of inhibitors, and then added to 100 μL of cell culture medium.
4. The resultant solution was added to 100 μL of JC-1 staining working solution at a 1:1 ratio, gently mixed thoroughly, and then incubated at 37 °C in a cell incubator for 20 min.
5. During incubation, an appropriate amount of JC-1 Buffer (1X) was prepared as follows: Each 1 mL of JC-1 Buffer (5X) was added with 4 mL of distilled water. The prepared solution was placed in an ice bath.
6. After incubation at 37 °C, the culture medium was removed by aspiration. The cells were gently rinsed twice using JC-1 buffer (1×).
7. The resultant cells were added to 100 μL of cell culture medium.
8. Measurement of fluorescence intensity using a microplate reader: For green fluorescence monomers, the excitation wavelength was set at 490 nm, and the emission wavelength was set at 525 nm. For red fluorescence polymers, the excitation wavelength was set at 540 nm, and the emission wavelength was set at 590 nm. The relative change in MMP content was calculated as the ratio of red fluorescence intensity to green fluorescence intensity multiplied by 100%. The measurement procedure was repeated at least three times.

#### 2.4.2 Determination of ATP content in granulosa cells

**FIGURE 1:**
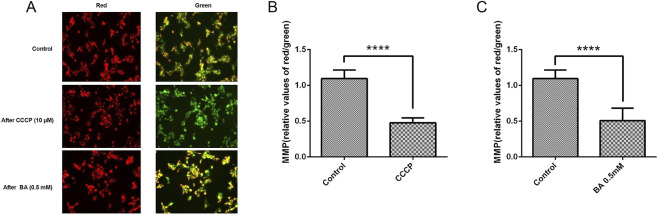
**(A)** Fluorescence microscopy: GCs were treated with CCCP (10 µM), BA (0.5 mM) for 20 min; as the MMP declined the intensity of red fluorescence decreased to green fluorescence. **(B,C)** The effect of CCCP (10 µM) and BA (0.5 mM) on MMP in mGCs. The bar graph represents the MMP fluorescence intensity. Mean values ±standard error of the mean (SEM) denote the MMP levels in 2 × 10^4^ mGCs. Student’s t-test for the two groups was used for statistical analysis (****P< 0.0001). The assay was repeated three times.

1. The granulosa cell suspension was seeded into 6-well culture plates at a cell density of 2 × 10^5^ cells per well and a suspension volume of 2 mL per well. After incubating at 37 °C in a 5% CO_2_ environment for 24 h, cell adhesion to the well was confirmed, and the culture medium from the wells was discarded. The granulosa cells were divided into three groups: the control group, the CCCP group, and the BA (0.5 mM) group. After the treatment of GCs with glycolysis inhibitor BA, the ATP content was signiffcantly reduced in all GCs, especially when the concentration of BA is 2.5 mM, this concentration leads to an increase in cell mortality rate, Therefore, it was not chosen.
2. A culture medium containing mitochondrial inhibitor CCCP (10 μM) and glycolysis inhibitor BA (0.5 mM) was added to each well at a volume of 100 μL. The culture medium in each well was discarded after 20 min of the drug reactions. Each well was filled with 200 μL of lysis buffer to lyse the cells.
3. The lysed suspension was centrifuged at 12,000 g and 4 °C for 5 min. Subsequently, the lysed suspension supernatant was transferred onto ice for subsequent determination of ATP levels.
4. Preparation of standard solutions: The ATP standard solution was thawed on an ice bath and diluted to concentrations of 0.01, 0.03, 0.1, 0.3, 1, 3, and 10 µM using ATP detection lysis buffer.
5. Preparation of the ATP detection working solution: A total of 100 μL of ATP detection working solution was required for each detected well. First, the ATP detection reagent was thawed in an ice bath. The stock solution was diluted using ATP detection reagent diluent at a ratio of 1:9. The prepared solution was stored in an ice bath for subsequent determination of ATP levels.
6. Determination of ATP concentration: a. 100 μL of ATP detection working solution was added into each well to be tested and left at room temperature for 3–5 min b. Each well to be tested was filled with 20 µL of sample solution and standard solution (the volume of the sample solution added was adjusted according to the ATP concentration of the sample). After the addition, the solution in the wells to be tested was quickly mixed with a micropipette. The fluorescence signal intensity of each well was measured using a chemiluminescence detector, ensuring at least a 2-s interval before each measurement. This measurement procedure was repeated three times for each sample. The detection process was repeated at least three times (Figure 1A).
7. The ATP concentration in the sample was computed based on the standard curve.

#### 2.4.3 Detection of reactive oxygen levels in granulosa cells

Principle: The detection method relies on the non-fluorescent property of 2′,7′-dichlorodihydrofluorescein diacetate (DCFH-DA) and its ability to freely pass through cell membranes. After entering the cells, DCFH-DA is hydrolyzed by intracellular esterases to generate DCFH. The hydrolyzed DCFH loses its capability to penetrate the cell membrane; thus, it is loaded as a probe within the granulosa cells.

DCFH, in its non-fluorescent form, is oxidized by intracellular ROS to generate DCF, which emits green fluorescence. The level of ROS within the cells can be determined based on the intensity of the green fluorescence.

The fluorescence signal intensity of each well was measured using a chemiluminescence meter at the maximum excitation wavelength of 480 nm and the maximum emission wavelength of 525 nm.

1. Probe preparation: DCFH-DA was diluted using serum-free culture medium at a ratio of 1:1000 to a final concentration of 10 μmol/L before loading the probe.
2. The granulosa cell suspension was seeded into a 96-well culture plate at a cell density of 2 × 10^4^ cells per well and a suspension volume of 100 μL per well. After incubating at 37 °C in a 5% CO_2_ environment for 24 h, cell adhesion to the well was confirmed, and the culture medium from the wells was discarded. The granulosa cells were divided into three groups: the control group, the CCCP group, and the BA (0.5 mM) group.
3. A culture medium containing mitochondrial inhibitor CCCP (10 μM) and glycolysis inhibitor BA (0.5 mM) was added to each well at a volume of 100 μL. This medication treatment process lasted for 20 min. Probe loading: The culture medium was removed from the wells by aspiration. Subsequently, each well was filled with 100 μL of DCFH-DA working solution. The granulosa cells were incubated for 30 min at 37 °C in a 5% CO_2_ dark environment.
4. Cell rinsing: The cells were gently rinsed once with serum-free culture medium.
5. Detection: The green fluorescence intensity, which indicates the relative absorbance value of the ROS level, was measured with a chemiluminescence meter at an excitation wavelength of 488 nm and an emission wavelength of 525 nm. The detection was repeated at least three times.

### 2.5 Detecting glucose consumption rate in granulosa cells treated with CCCP inhibitors using the glucose assay kit

1. The granulosa cell suspension was seeded into 96-well culture plates at a cell density of 1 × 10^6^ cells per well and a suspension volume of 100 μL per well. Blank control group and treatment group have 12 technical wells. Each donor cell is set up with 12 wells (technical replicates), and the blank control group and treatment group (control group, CCCP group) are set up according to this setting. The experiment is repeated 3 times, and the average of the wells is taken as the single sample result to reduce experimental errors. After incubating at 37 °C in a 5% CO_2_ environment for 24 h, cell adhesion to the well was confirmed. The granulosa cells were divided into two groups: the control group and the CCCP group.
2. Culture mediums in the wells were discarded. One group was randomly selected and supplemented with culture medium containing the mitochondrial inhibitor CCCP (10 μM). Culture of these cells was continued for 24, 48, and 72 h.
3. The cell culture medium was collected after 24, 48, and 72 h of drug administration. The glucose content was measured using a glucose assay kit (Jiancheng, Nanjing, China), according to the manufacturer’s protocol. And use glucose standards with known concentrations (0, 0.1, 0.2, 0.5, 1.0, 2.0 mmol/L) to draw standard curves for calibration. The supernatants were stored at −80 °C for the glucose test. The samples were measured with a microplate reader at an absorbance of 505 nm.
4. Glucose consumption was determined by comparing the glucose level in the original culture medium with the culture medium.
5. Glucose consumption rates expressed as mmol/10^6 cells/h for each time point.

### 2.6 Detecting the proliferation activity of mGCs treated with CCCP inhibitors using the cell counting kit-8 (CCK-8) assay

1. The granulosa cell suspension was seeded into 96-well culture plates at a cell density of 2 × 10^4^ cells per well. After culturing at 37 °C in a 5% CO_2_ environment for 24 h, when the cells adhered to the well, the granulosa cells were divided into two groups: the control group and the CCCP group.
2. Culture mediums in the wells were discarded. One group was randomly selected and supplemented with culture medium containing the mitochondrial inhibitor CCCP (10 μM) at a volume of 100 μL per well, and the culture was continued for 24, 48, and 72 h.
3. Each well was added with 10 µL of CCK-8 solution after 24, 48, and 72 h of cell culture.
4. The culture plate was placed in a 37 °C, 5% CO_2_ incubator for 3 hours.
5. The absorbance of each well was measured at 450 nm using a microplate reader. Each sample underwent three repeated measurements. The detection was repeated at least three times.
6. The following formula was used for calculating cell viability (%):

$$\begin{array}{ll} & \text{Cell viability }\left(\%\right)=\left[A\left(\text{with drug treatment}\right)\;–\;A\right.\\ & \;\left.\left(\text{blank}\right)\right]/\;\left[A\left(0\text{ drug treatment}\right)\;–\;A\left(\text{blank}\right)\right]\times 100 \end{array}$$

### 2.7 Detection of glycolysis-related genes using Quantitative Real-time polymerase chain reaction (qRT-PCR)

#### 2.7.1 Cell RNA extraction

1. The granulosa cell suspension was seeded into 6-well culture plates at a cell density of 2 × 10^5^ cells per well and a suspension volume of 2,000 μL per well. After culturing at 37 °C in a 5% CO_2_ environment for 24 h, cell adhesion to the well was confirmed. The granulosa cells were divided into two groups: the control group and the CCCP group.
2. The two groups were treated with CCCP drugs, and cultured for 24, 48, and 72 h. The culture medium in the wells was discarded. Each well was filled with 1 mL of Trizol lysis reagent, placed at room temperature for 2 min, and then pipetted up and down repeatedly. The lysis reagent was then collected from each well into 1.5-mL Eppendorf (EP) tubes. The tubes were then placed on ice and kept still for 5 minutes. The EP tubes were added with 200 μL of chloroform, vigorously shaken for 30 s, placed on ice for 10 min, and centrifuged at 12,000 r/min for 15 min at 4 °C.
3. The supernatant in the EP tubes was then carefully transferred to another 1.5-mL centrifuge tube using a pipette. The 1.5-mL centrifuge tube was filled with an equal volume of isopropanol, gently inverted for a thorough mix until the layers disappeared, and then placed on ice for 10 min. The 1.5-mL centrifuge tube was then centrifuged at 12,000 r/min for 10 min at 4 °C. The supernatant was discarded, and 1 mL of 75% ethanol was added into the 1.5-mL centrifuge tube. The resultant 1.5-mL centrifuge tube was centrifuged at 12,000 r/min for 5 min at 4 °C. The supernatant was discarded.
4. The extracted RNA underwent air drying for 30 min. Afterwards, an appropriate amount of 0.1% diethyl pyrocarbonate (DEPC) solution was added to dissolve the RNA that had precipitated at the bottom of the tube. Subsequently, the RNA content was measured using an ultraviolet (UV) spectrophotometer.

#### 2.7.2 The methods for RNA quantification, reverse transcription, and amplification were the same as those described in the initial part of the experiment

##### 2.7.2.1 PCR primer sequences

Designed primers targeting the genes—GLUT1, LDHA, PFKP, HIF-1α, and β-actin—were tested using the qRT-PCR.

Primers were designed based on literature references and underwent verification. The primers were synthesized by Shanghai Generay Biotech Co., Ltd. The primer sequences used were as follows:

GLUT1 F 5′-ccagctgccattgccgtt-3′
  R 5′-gacgtagggaccacacagttgc-3′
LDHA F 5′-tgcacccagatttagggactgat-3′
  R 5′-cccaggatgtgtagcctttgag-3′
PFKP F 5′-aggcgatggacgagaggagat-3′
  R 5′-tgatggcaagtcgcttgtag-3′
HIF-1α F 5′-catcagctatttgcgtgtgagga-3′
  R 5′-agcaattcatctgtgctttcatgtc-3′
  β-actin F 5′-tgacgtggacatccgcaaag-3′
  R 5′-ctggaaggtggacagcgagg-3′

##### 2.7.2.2 qRT-PCR reaction conditions

Pre-denaturation was performed for 10 min at 95 °C. The cycling parameters included denaturation at 95 °C for 15 s; annealing at 55 °C–60 °C for 20 s; and extension at 72 °C for 15 s. A total of 45 cycles were conducted. This procedure was repeated three times for each sample. The amplification efficiency of all primers was validated, and the expression of the reference gene β-actin was confirmed to be stable across experimental groups.

##### 2.7.2.3 Analysis of PCR results

The cycle threshold (CT) value of each reaction well was recorded after the PCR reaction. β-actin Ct mean is 14.26 ± 0.24. The calculation was carried out as per the formula: 2^-△△CT^. The final results were expressed as the mean ± standard deviation ( $\bar{x}$ ± s).

### 2.8 Statistical analysis

All statistical analyses were performed using SPSS 24.0, with data presented as mean ± standard deviation (mean ± SD). Group comparisons were conducted using Student’s t-test for two-group comparisons and one-way analysis of variance (one-way ANOVA) for multiple-group comparisons to determine if there were significant differences among the different treatment groups. For results showing significant differences by ANOVA, a P value less than 0.05 was considered statistically significant (P < 0.05).

## 3 Results

### 3.1 Granulosa cell MMP

The MMP levels in mGCs were significantly reduced following treatment with the mitochondrial inhibitor CCCP (10 μM). These levels were significantly lower in comparison to those in the control group (*P* = 0.03) (Figure 1B).

After treatment with the glycolysis inhibitor BA (0.5 mM), the MMP levels in mGCs were significantly reduced and significantly lower in comparison to those in the control group (*P* = 0.03) (Figure 1C).

### 3.2 ROS content in granulosa cells

**FIGURE 2:**
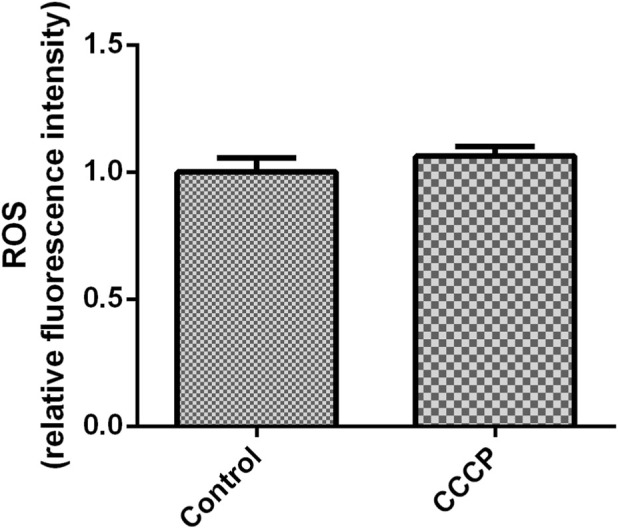
The effect of CCCP (10 µM) on ROS in mGCs. The bar graph represents ROS fluorescence intensity. Mean values ±SEM indicate ROS levels in 2 × 10^4^ mGCs. Student’s t-test for the two groups was used for statistical analysis (*P* = 0.06). The assay was repeated three times.

mGCs were tested for the ROS level after treatment with the mitochondrial inhibitor CCCP (10 μM). The results showed that the CCCP group had a slightly higher ROS level than the control group, but this significance was not statistically significant (*P* > 0.05) (Figure 2).

**FIGURE 3:**
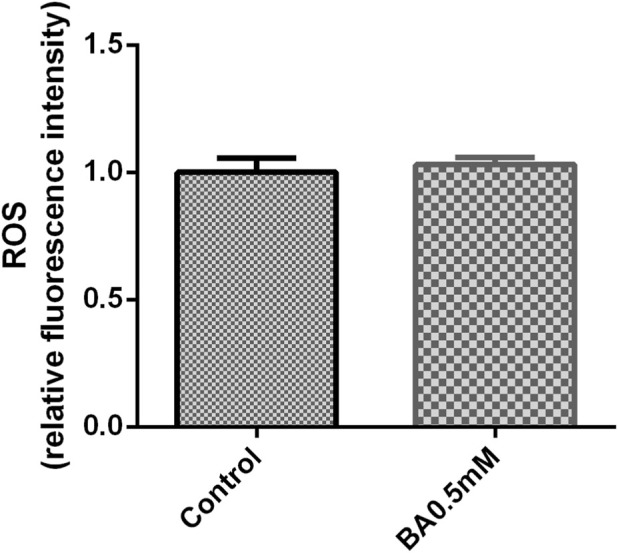
The effect of BA (0.5 mM) on ROS in mGCs. The bar graph represents ROS fluorescence intensity. Mean values ±SEM denote ROS levels in 2 × 10^4^ mGCs. Student’s t-test for the two groups was used for statistical analysis (*P* = 0.26). The assay was repeated three times.

When mGCs were tested for the ROS level after treatment with the glycolysis inhibitor BA (0.5 mM), the BA (0.5 mM) group had a slightly higher ROS level than the control group, but this significance was not statistically significant (*P* > 0.05) (Figure 3).

### 3.3 ATP content in granulosa cells

**FIGURE 4:**
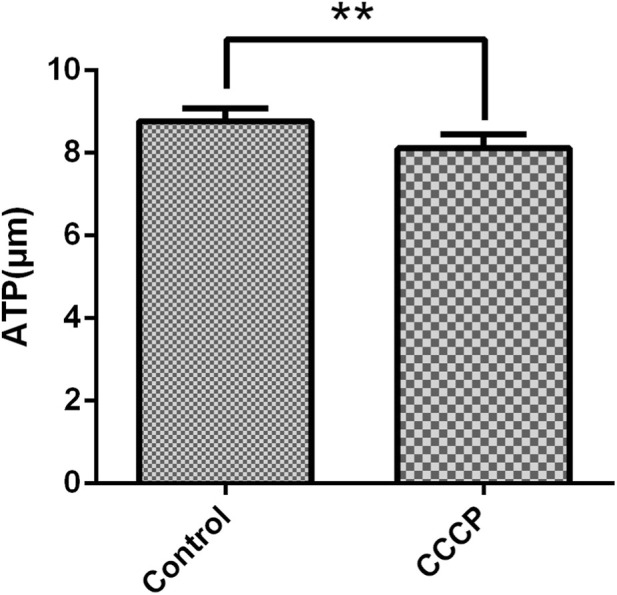
The effect of CCCP (10 µM) on ATP in mGCs. The bar graph represents the ATP fluorescence intensity. Mean values ±SEM represent ATP levels in 2 × 10^4^ mGCs. Student’s t-test for the two groups was used for statistical analysis (**P = 0.001). The assay was repeated three times.

With respect to ATP levels in mGCs after treatment with the mitochondrial inhibitor CCCP (10 μM), the CCCP group showed a decrease in the ATP content, which was significantly lower than that in the control group (*P =* 0.001) (Figure 4).

**FIGURE 5:**
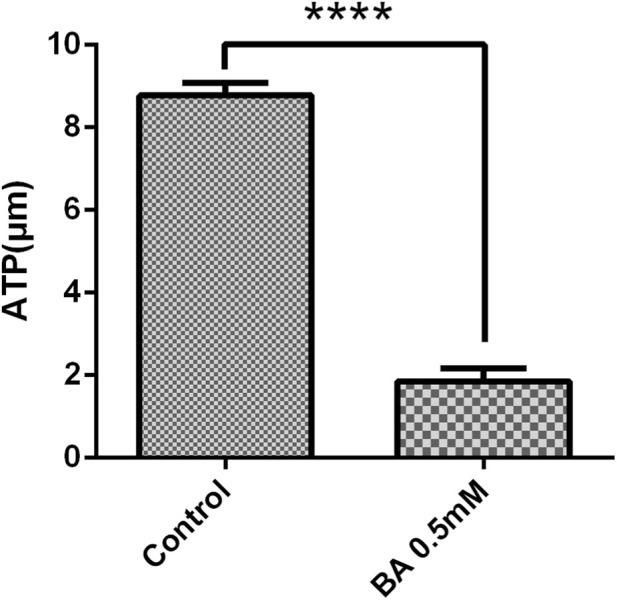
The effect of BA (0.5 mM) on ATP in mGCs. The bar graph represents the ATP fluorescence intensity. Mean values ±SEM represent ATP levels in 2 × 10^4^ mGCs. T-test for the two groups was used for statistical analysis (****P < 0.001). The assay was repeated three times.

Testing mGCs for the ATP level after treatment with the glycolysis inhibitor BA (0.5 mM) revealed that the BA (0.5 mM) group showed a decrease in ATP, which was significantly lower than that in the control group (*P* < 0.0001) (Figure 5).

### 3.4 CCCP-induced HIF-1α and glycolysis-related gene expression in mGCs

**FIGURE 6:**
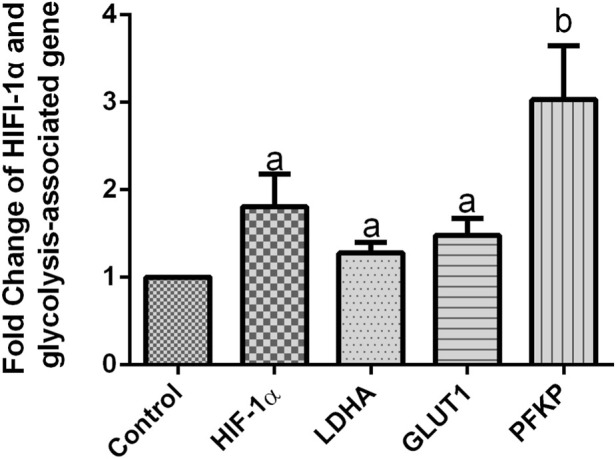
The effect of CCCP on HIF-1α and glycolysis-related genes in mGCs, statistical analysis was conducted using a one-way ANOVA (a,b: *P* < 0.05). The assay was repeated three times.

mGCs were treated with the mitochondrial inhibitor CCCP (10 µM). The mitochondrial oxidative respiratory chain function was interfered with using a mitochondrial dysfunction model. The results showed that the expression of HIF-1α mRNA in mGCs treated with the mitochondrial inhibitor CCCP (10 µM) was 1.8 times higher than that of the control group, with a statistically significant difference (*P* < 0.05). The mRNA expression levels of GLUT1 and LDHA were upregulated in mGCs. Compared with those in the control group, GLUT1 mRNA, LDHA mRNA, and PFKP mRNA in mGCs were upregulated by 1.4, 1.2, and 3.0 times, respectively. These differences were statistically significant (*P* < 0.05) (Figure 6).

### 3.5 The effect of mitochondrial function inhibition on the glycolytic function of mGCs

**FIGURE 7:**
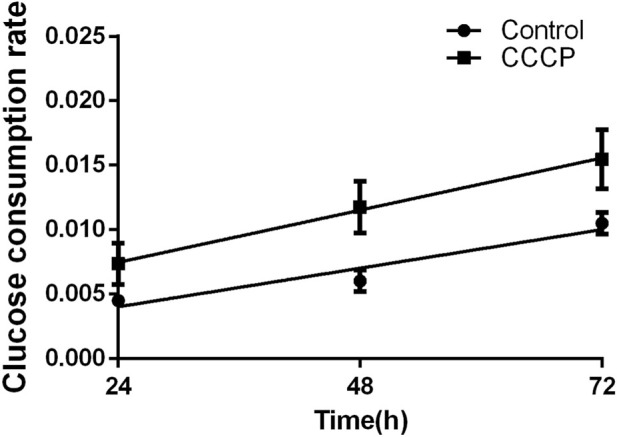
Changes in mGC glucose consumption at 24, 48, and 72 h following CCCP treatment. The glucose consumption rate was compared between the CCCP group and the control group. Data are presented as the mean ± standard deviation of 58 independent donors (N = 58). Glucose consumption rates are expressed as mmol/10^6 cells/h. Statistical analysis was conducted using one-way ANOVA (P < 0.05).

The glycolytic function of mGCs was evaluated following treatment with the mitochondrial inhibitor CCCP (10 µM) and culture for 24, 48, and 72 h. The results showed that glucose consumption of mGCs was significantly higher in the CCCP (10 µM)-treated group after 24, 48, and 72 h compared with that in the control group. Glucose uptake by mGCs increased progressively with prolonged culture time, with the highest glucose consumption observed at 72 h post-treatment. The differences were statistically significant (*P* < 0.05) (Figure 7).

### 3.6 The effect of mitochondrial function inhibition on the viability of mGCs

**FIGURE 8:**
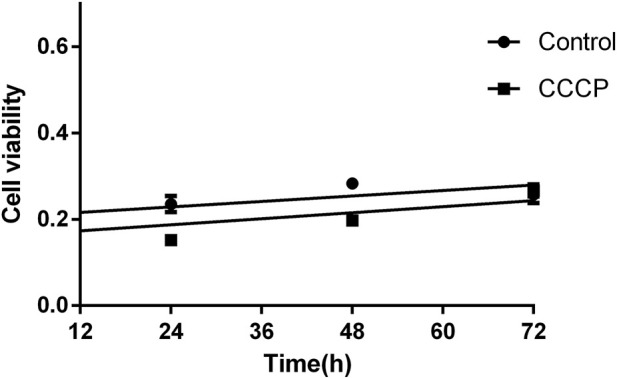
Cell viability changed in mGC at 24, 48, and 72 h following CCCP treatment. Cell Counting Kit-8 (CCK8) confirmed and compared the cell growth between the CCCP group and the control group, statistical analysis was conducted using a one-way ANOVA (*P* < 0.05).

Following treatment with the mitochondrial inhibitor CCCP (10 µM) and culture for 24, 48, and 72 h, the viability of mGCs was measured using the CCK-8 assay. It was found that mGC viability gradually increased with prolonged culture time, with statistically significant differences (*P* < 0.05). However, compared to the control group, the viability of mGCs cultured for 24, 48, and 72 h was significantly lower, with statistically significant differences (*P* < 0.05). Notably, after 72 h of culture, there was no statistically significant difference in cell viability between the CCCP and control groups (*P* > 0.05) (Figure 8).

## 4 Discussion

The findings in this study indicate that the mitochondrial function of ovarian granulosa cells decreased after human primary ovarian granulosa cells were treated with mitochondrial and glycolysis inhibitors, as evidenced by a significant reduction in MMP and ATP levels and a slight increase in ROS levels. Cell function tests further confirmed that the inhibition of mitochondrial function resulted in an increase in the glycolytic activity of ovarian granulosa cells, accompanied by enhanced granulosa cell viability.

The ATP content of oocytes is closely associated with human embryogenesis and increased implantation rates (Van Blerkom et al., 1995). To meet the energy demands of the oocyte, the number of mitochondria within it can dramatically increase to 10,000, along with an increase in their activity. However, oocytes lack the ability to synthesize glucose-metabolizing enzymes and have a relatively low capacity to directly utilize glucose as an energy substrate. Therefore, to ensure an adequate supply of energy during oocyte maturation, most glucose is first glycolyzed into pyruvate by granulosa cells (Hoque et al., 2021; Yuan et al., 2016). Pyruvate is then transported to the mitochondria of the oocyte via the monocarboxylic acid transport system (Xie et al., 2016), providing energy substrates for the oocyte’s energy production (Orisaka et al., 2009; Wen et al., 2020; Lowe et al., 2019). Therefore, the stability of granulosa cell functions such as division and proliferation is crucial for providing the necessary energy support for oocyte growth and homeostasis (Guo et al., 2024; Oi et al., 2015).

Based on our research, we identified two energy metabolism pathways during follicular development and maturation in granulosa cells—oxidative phosphorylation and glycolysis. These metabolic pathways are involved in a series of physiological activities, such as granulosa cell proliferation, which in turn maintains the stability of the follicular microenvironment. Disrupted energy metabolism can reduce oocyte quality and the developmental potential of subsequent embryos (Köse and Nazıroğlu, 2014; Gilchrist et al., 2004), but the specific regulatory mechanisms remain unclear. Studies have reported a close relationship between the functionality of granulosa cell mitochondria and oocyte quality, as well as overall female fertility (Vij et al., 2018; Wang et al., 2010; Niu et al., 2021). Enhancing the mitochondrial function of granulosa cells can effectively improve oocyte activity and the success rate of ART. A more in-depth study and analysis of granulosa cell mitochondrial function reveals that stable mitochondrial function may be involved during a critical period of energy pattern switching during follicular development.

We cultured human mural granulosa cells *in vitro* and introduced mitochondrial inhibitors to simulate changes in mitochondrial function, thereby establishing a model of mitochondrial dysfunction. Subsequent analyses revealed a decrease in MMP and ATP levels and an slightly increase in ROS levels within the cells, suggesting a decrease in mitochondrial metabolic activity within granulosa cells. After treated with glycolysis inhibitors, we also found the same results, which introduced that glycolysis is not only plays a critical role in GC energy metabolism, but also influences mitochondrial oxidative metabolism to some extent. The source of increased ROS but not significant upward trend might be due to the impaired mitochondrial OXPHOS in GCs. It is speculated that this is a signal representing the energy imbalance state of granulosa cells.

Additional analyses of the glucose metabolism function of granulosa cells in this study showed an increased expression of HIF-a, GLUT1, LDHA, and PFKP genes encoding the rate-limiting enzymes of the glycolysis pathway in granulosa cells. This upregulation was accompanied by decreased glucose content and increased glucose consumption in the culture medium. Ernst EH and Zhang X et al. found glycolysis-related proteins were significantly increased in granulosa cells (Ernst et al., 2018; Zhang et al., 2022). HIF-a is the master regulator of cellular response to hypoxia. Under hypoxia conditions, cells switch to the oxygen-independent metabolic pathway, and produce ATP using the glycolysis pathway (Stark et al., 2015; Laganà et al., 2016). This process is also essential for higher quality oocytes and embryonic development. GLUT1, located on the plasma membrane of various cells, has a high facilitates glucose absorption into cells. LDHA is a glycolytic enzyme whose primary function is to catalyze the oxidation of lactate to pyruvate, transferring hydrogen to NAD to form NADH. PFKP is the rate-limiting enzyme of glycolysis, regulating a key step in the glycolysis pathway. Studies have revealed that when the mitochondrial function of granulosa cells changes, the energy metabolism of granulosa cells gradually shifts to the glycolysis pathway (Gannon et al., 2013).

As revealed by the CCK-8 assay targeting granulosa cells cultured continuously for 24, 48, and 72 h, following oxidative stress stimulation, human ovarian granulosa cells showed an increase in the expression of glycolysis-related genes, enhanced glycolytic enzyme activity, and an improvement in cell efficiency in utilizing glucose, thereby increasing the viability of granulosa cells. These findings suggest that the energy metabolism mode of human ovarian granulosa cells shifts to adapt to the survival needs of cells. The transition from mitochondrial oxidative phosphorylation to glycolysis promotes late-stage cellular development.

The above results further confirm that changes in mitochondrial function affect the balance of intracellular energy metabolism. The compensatory increase in glycolytic function of granulosa cells represents an adaptive adjustment strategy made by cells in response to changes in the external environment. Such cell adjustment is more conducive to maintaining cell energy balance and promoting cell viability. Animal studies in dairy cows have shown that, under hypoxic conditions, the metabolic shift from oxidative phosphorylation to glycolysis during folliculogenesis plays a key role in the rapid proliferation of ovarian granulosa cells (Shiratsuki et al., 2016). By studying the mitochondrial and glycolytic functions of human granulosa cells, it is further suggested that abnormal energy metabolism of granulosa cells is involved in the abnormal process of follicular development. Our research is expected to improve the IVF-ET outcomes of infertile women caused by ovulation disorders. In subsequent research, we will investigate the significant role of the hypoxic metabolic pathway in the metabolic conversion of ovarian granulosa cells and the development of follicles. Although our study noted a slight increase in ROS levels following mitochondrial inhibition, the implications of ROS in cellular signaling, oxidative stress, and apoptosis in granulosa cells require further investigation. Given ROS’s critical role in cell fate, understanding how elevated ROS levels affect granulosa cell function over time is essential. Our research also has limitation, due to the extreme fragility of primary human granulosa cells, we found that prolonged handling for Annexin-V/PI flow cytometry caused substantial cell detachment and loss, yielding unreliable data, which precluded us from distinguishing true cellular adaptation from selective survival.

## 5 Conclusion

In conclusion, our results revealed that the administration of mitochondrial inhibitors significantly decreased MMP and ATP levels in human ovarian granulosa cells. And there was an increase in the expression of genes related to glucose transport and uptake. These findings indicate the presence of two energy metabolism ways—oxidative phosphorylation and glycolysis—in human granulosa cells during the late stage of follicular development and maturation. However, the lack of validation at the protein level such as WB represents a major limitation. We need further research to confirm that both metabolic pathways jointly contribute to maintaining a stable follicular microenvironment. Future studies will assess mitochondrial and glycolytic functions in ovarian granulosa cells from patients with diminished ovarian reserve to further verify that abnormal energy metabolism contributes to follicular maturation disorders. We will screen upstream regulatory genes and validate energy metabolism functions in human ovarian granulosa cell lines via gene knockdown or overexpression. These studies are expected to provide theoretical support for improving clinical outcomes of infertility related to follicular dysfunction.

## Data Availability

The original contributions presented in the study are included in the article/supplementary material, further inquiries can be directed to the corresponding author.
